# Area and Bandwidth Enhancement of an n^+^/p-Well Dot Avalanche Photodiode in 0.35 μm CMOS Technology

**DOI:** 10.3390/s23073403

**Published:** 2023-03-23

**Authors:** Seyed Saman Kohneh Poushi, Bernhard Goll, Kerstin Schneider-Hornstein, Michael Hofbauer, Horst Zimmermann

**Affiliations:** Institute of Electrodynamics, Microwave and Circuit Engineering, Vienna University of Technology, 1040 Vienna, Austria

**Keywords:** CMOS-integrated dot avalanche photodiode, multi-dot APD, radial charge collection, active area enlargement, bandwidth enhancement

## Abstract

This paper presents a CMOS-integrated dot avalanche photodiode (dot-APD) that features a small central n^+^/p-well hemispherical cathode/p-well structure circularly surrounded by an anode ring. The dot-APD enables wide hemispherical depletion, charge collection from a large volume, and a small multiplication region. These features result in a large light-sensitive area, high responsivity and bandwidth, and exceptionally low junction capacitance. The active area can be further expanded using a multi-dot structure, which is an array of several cathode/p-well dots with a shared anode. Experimental results show that a 5 × 5 multi-dot APD with an active area of 70 μm × 70 μm achieves a bandwidth of 1.8 GHz, a responsivity of 9.7 A/W, and a capacitance of 27 fF. The structure of the multi-dot APD allows for the design of APDs in various sizes that offer high bandwidth and responsivity as an optical detector for various applications while still maintaining a small capacitance.

## 1. Introduction

Because of the inherent gain, using linear-mode avalanche photodiodes (APDs) instead of standard pin photodiodes has become attractive in many optical systems where weak optical signal detection is required [1,2,3,4,5,6,7]. The use of a CMOS process for the production of integrated APDs with electronic circuitry for read-out and signal processing reduces the influence of parasitic effects, and furthermore offers a cost-effective production. Common design approaches towards CMOS-integrated APDs are based on planar n^+^/p-well and p^+^/n-well junctions. Refs. [8,9,10,11,12,13,14,15] present APDs with a thin combined absorption and multiplication region in the order of hundreds of nanometers thickness. These APDs provide a high bandwidth but suffer from low responsivity at long wavelengths (red and near-infrared light) due to their thin detection zone. Refs. [14,15] reported APDs that achieve a bandwidth of 8.4 GHz and 12 GHz while their maximum responsivities are 0.56 A/W and 0.03 A/W, respectively, at a wavelength of 850 nm.

In order to achieve a higher responsivity, the so-called reach-through APD was presented, which possesses a separate space-charge region with a larger thickness [16,17,18,19,20]. A 12 μm thick absorption region was present in [17,19,20]. In these APDs, the photo-generated electrons in the thick absorption zone drift upwards to the multiplication zone and can trigger the avalanche process, resulting in a high responsivity. However, carrier drift time in their thick absorption region generally limits the bandwidth. Based on the reach-through concept, Ref. [17] presented an APD with the responsivity and bandwidth of 20.5 A/W and 850 MHz at 670 nm. Refs. [19,20] used the lateral well modulation-doping technique to improve the bandwidth by manipulating the electric field distribution within the structure.

One of the main drawbacks of the planar structures is that the photo-sensitive area is proportional to the area of the p/n junction. In fact, since the electric field is limited to below the p/n region, only the carriers generated in this area have a chance to cause impact ionization by electrons drifting to the multiplication region. Therefore, to increase the sensitive area, the p/n junction area has to be expanded, which leads to an increase in the junction’s capacitance. Consequently, these APDs typically exhibit the trade-off between detector capacitance and the detection area. In addition, the proportionality of the active area to the p/n junction area results in a limited fill-factor defined as the ratio of the photo-sensitive area to the whole diode area. Moreover, the necessity of a guard ring to avoid premature edge breakdown in such structures causes further fill-factor degradation. This degradation is more noticeable in the scaled-down APD, where the dimensions of the guard ring are comparable to the photos-sensitive area dimensions. Accordingly, the scalability of such APDs to be used in multi-pixel detectors is an issue [21,22,23].

Guiding carriers generated in the peripheral volume towards the multiplication region can provide a near-unity fill-factor and overcome the scalability issue. A current-assisted avalanche photodiode is presented in [24] consisting of two electrodes at the surface with different potentials to collect photo-generated carriers in the peripheral volume. This APD achieved a near-unity fill-factor and a responsivity of 13 A/W, but its maximum bandwidth reported was 275 MHz at *λ* = 830 nm. Ref. [25] used the field-line crowding concept with a small n^+^/n-well structure to form a distributed electric field over the whole diode volume to guide the photo-generated carriers in the peripheral volume. This device was realized in 0.18 µm CMOS with a 24 µm thick low-doped epitaxial layer. A maximum bandwidth of 1.6 GHz and a responsivity of R = 32 A/W at *λ* = 675 nm were achieved for an APD with a radius of 19 μm while having a near-unity fill factor.

This paper presents a dot avalanche-based APD that provides a near-unity fill factor and 9.7 A/W responsivity while achieving 1.8 GHz bandwidth at *λ* = 675 nm. The single dot structure possesses a small hemispherical n^+^/p-well structure at the center. A spherically uniform high electric field is formed around the n^+^/p-well junction, which serves as the multiplication region inside the p-well, and a weaker electric field extends radially throughout the diode to guide charge carriers from the entire diode volume towards the multiplication region. For applications that require a larger light-sensitive area, a multi-dot structure is proposed to increase the active area, which is a combination of several cathode/p-well dots with connected cathodes and with a shared anode. In the next sections, the design approach will be discussed in detail.

## 2. Single-Dot APD

**Figure 1:**
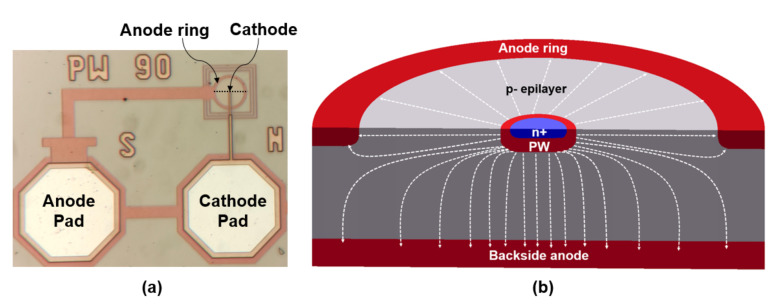
(**a**) Top view and (**b**) 3d schematic cross-section (not to scale) along the dashed line in sub-figure (**a**) of single-dot n^+^/p-well APD fabricated in 0.35 μm CMOS technology. The cathode radius is 0.9 μm and the total diode radius is 14 μm.

Figure 1 shows the top view (a) and a cross-section (b) of the single-dot APD (SD-APD) fabricated in the 0.35 μm CMOS modular optical sensor technology platform (XO035) of X-FAB semiconductor foundries. A semi-hemispherical high-doped n^+^ region embedded in a half-sphere p-well (PW) region as the multiplication region forms the cathode of this APD. The cathode is circularly surrounded by a p^+^ and p-well as an anode, which all are formed on a lightly p-doped epitaxial layer (p- epi) with a doping concentration of ∼$2\times 10^{13}$ cm${}^{-3}$ and a thickness of ∼12 μm. The p^+^ substrate is also used as a backside anode as it is available at the back side of the chip (because the wafers were thinned by mechanical grinding) and can be connected to the anode supply.

In the SD-APD, unlike the planar structures that only have a vertical electric field, the electric field extends radially throughout the diode, which is used for vertical and peripheral charge collection. TCAD simulation is performed to study the electric field distribution inside the structure. According to the cylindrically symmetric geometry of the structure, two-dimensional (2d) simulations of half of the diode are carried out along one radius in the cylindrical coordinate system. In order to enhance the simulation accuracy and to optimize the simulation time, the mesh of grid points in the cathode region (n^+^/p-well junction) is refined, while it becomes coarser towards the substrate and anode ring.

**Figure 2:**
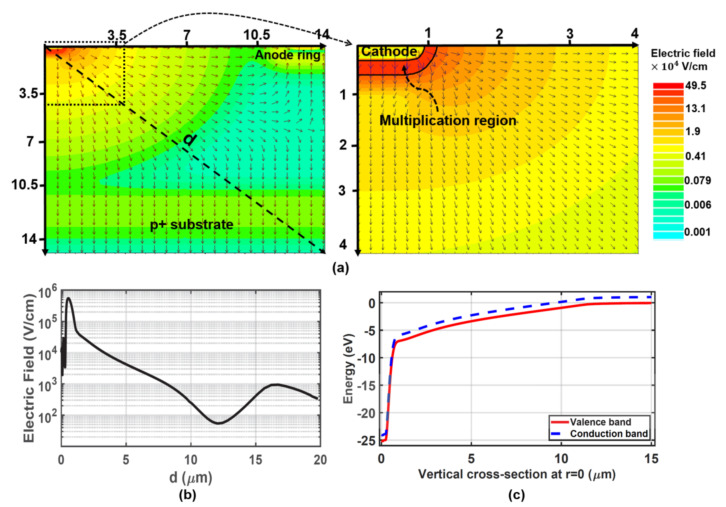
(**a**) 2d plot of the electric field distribution across the SD-APD obtained by TCAD simulations at the operating voltage of 24.5 V. Arrows indicate the local electric field direction. (**b**) Radial cross-section of the electric field along the dashed line (d) in the structure. (**c**) Energy band diagram along a vertical cross-section at r = 0 μm.

Figure 2a shows a 2d plot of the electric field in the SD-APD at an operating voltage of 24.5 V (breakdown voltage = 25.2 V) simulated using the SILVACO Atlas. It can be seen that a semi-hemispherical multiplication region with a high electric field strength of 4.95 × 10^5^ V/cm is formed at the junction of n^+^/p-well with a width of ∼0.4 μm at the critical field strength for impact ionization of 2 × 10^5^ V/cm. Based on TCAD simulation results and according to the design rules and doping profiles associated with the 0.35 μm XO035 CMOS technology, the n^+^ and p-well radii were set to 0.85 μm and 0.9 μm, respectively, to achieve a uniform high-field hemispherical distribution. It should be noted that the 0.85 μm size for the n+ region radius is the minimum size available to form a round n^+^ region based on the design rules and doping profiles of the 0.35 μm XO035 CMOS technology. Simulation results indicate that a p-well radius of less than 0.9 μm results in a non-uniform electric field distribution.

The electric field extends but lowers by moving radially away from the cathode towards the substrate, towards the anode ring and in a “diagonal” direction, as shown in Figure 2b. For example, at a “diagonal” distance of d = 10 μm from the origin of coordinates, the electric field is still ∼200 V/cm. Figure 2c shows the energy band diagram along a vertical cross-section at r = 0 μm. According to the energy band diagram when a photon is absorbed in the detection region, the generated electron and the hole are promptly separated by the electric field (drifting in opposite directions), and then, the electron drifts into the direction opposite to the electric field vector arrows towards the small multiplication region. This figure also shows that the reverse voltage of 24.5 V splits into about 7.5 V over the thick absorption region and 17 V over the multiplication region. In fact, due to the existence of the lateral component of the electric field, the peripheral carriers also have a chance to reach the multiplication region, where they can start impact ionization. Therefore, the whole diode area acts as the detection zone, which results in a large light-sensitive area. In addition, the drift-based carrier transport mechanism enhances the detector’s speed performance.

**Figure 3:**
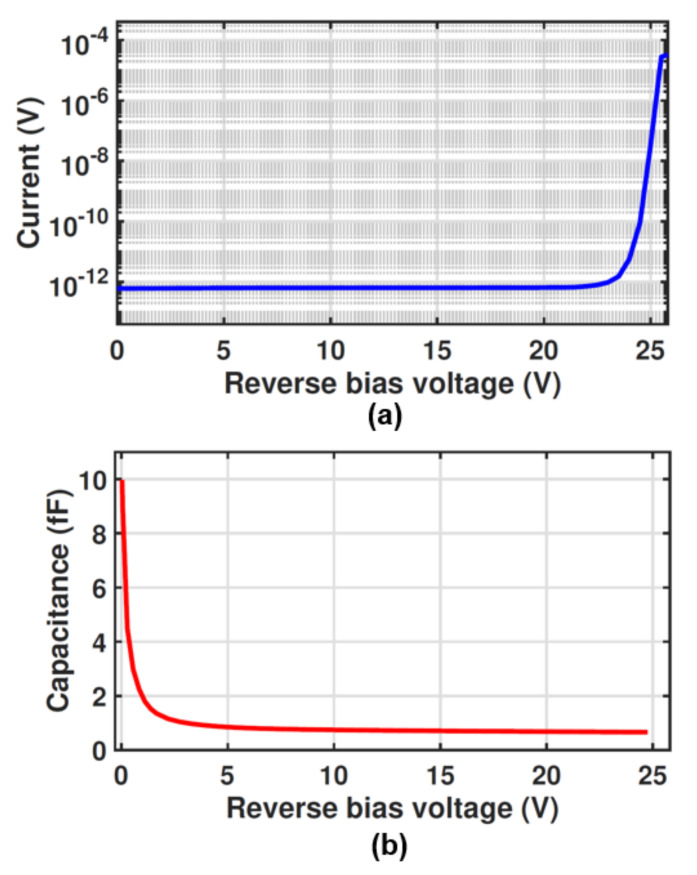
(**a**) Measured dark reverse characteristics and (**b**) Simulated capacitance of the SD-APD as a function of reverse bias voltage.

Furthermore, since the electric field penetrates deeply into the structure (e.g., the electric field in a depth of 10 μm at r = 0 is ∼400 V/cm), the detection zone extends down to the p^+^ substrate, which means the photo-generated carriers in the deep depth can drift towards the multiplication region and trigger an avalanche event. As a result, the large thickness of the detection region provides a high responsivity for long wavelengths. The responsivity and frequency response are assessed through TCAD simulations. The responsivity and bandwidth of 0.27 A/W and 1.25 GHz, respectively, have been obtained for the SD-APD. Such a concentrating electric field distribution causes a breakdown voltage of 25.2 V where the dark current reaches 1 μA [26] as can be seen in Figure 3a. However, the breakdown voltage varies as the radius of the p-well changes because the electric field distribution within the structure changes.

It is worth mentioning that the capacitance of the SD-APD is expected to be low due to the small area of the p/n junction. The capacitance of the SD-APD is evaluated using TCAD simulations as we cannot measure it due to the accuracy of our current equipment. Figure 3b presents the simulated capacitance of the SD-APD as a function of the reverse bias voltage. It can be seen that the capacitance is ∼10 fF at 0 V bias voltage and quickly drops to sub-Femto Farad values when increasing the reverse bias voltage. At the operating voltage of 24.5 V, the capacitance is 0.65 fF, which is significantly smaller than the typical values of a conventional planar CMOS APDs [17,27].

It is important to note that it is not possible to increase the size of the active area only by simply increasing the radius of the surface anode of the diode when a large active area is required (e.g., free space optical communications). Because, as previously shown (Figure 2), the electric field gradually decreases by moving radially away from the center, and therefore, at distances far from the center, the electric field is very weak or non-existent to drive carriers towards the cathode. Accordingly, the transfer mechanism is no longer carrier drift but carrier diffusion which decreases the responsivity and the bandwidth of the APD. The diffusion transport mechanism leads to a significant reduction in the bandwidth of the APD as the transit time of the photogenerated carriers to reach the cathode strongly increases. However, to increase the active area, a multi-dot structure is proposed, which can provide high responsivity and bandwidth, as detailed below.

## 3. Multi-Dot APD

**Figure 4:**
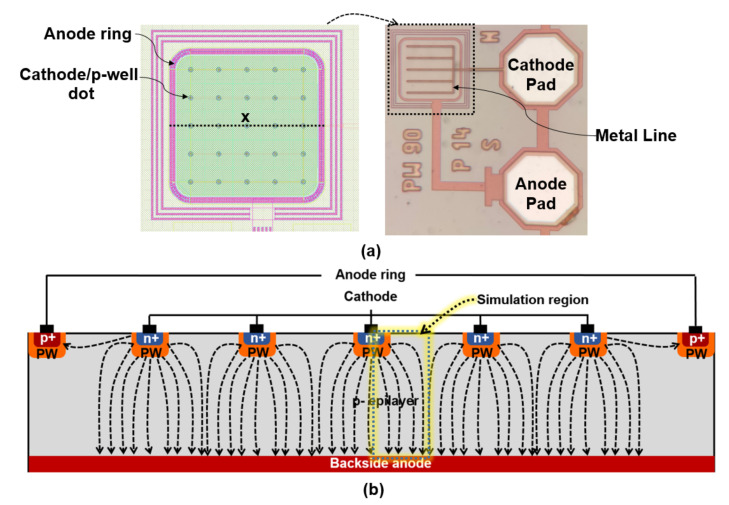
(**a**) Top view of the 5 × 5 array MD-APD fabricated in 0.35 μm CMOS technology. Left is the layout drawn in Cadence and right is a photograph of the fabricated chip. (**b**) is a schematic cross-section (not to scale) of the MD-APD along the dotted line (x) in sub-figure (**a**) left.

Figure 4 shows the top view (a) and a 2d schematic cross-section (b) of the multi-dot APD (MD-APD) fabricated in the XO035 CMOS technology. The MD-APD is an array (5 × 5) of single-dot cathode/p-well structures, where the single cathodes are connected with tracks in metal layer 4 with a minimum width of 0.6 μm as shown in Figure 4a. The p^+^ substrate is used as a shared backside anode. In addition, the dot cathode array is surrounded by a surface anode where the electric field of the boundary dots terminates, and defines the size of the diode. The APD is covered by the standard isolation and passivation stack to protect the fabricated device from environmental influences.

When the MD-APD is reversely biased, the hemispherical multiplication region is formed around each cathode dot. The area between the dots is fully depleted. The longest electric-field lines with the weakest electric field strength (between two dots) are high-lighted in Figure 4b. Therefore, the region under and between each cathode acts as a detection zone so that the photo-generated carriers are accelerated towards a cathode according to the local electric field direction. As a result, this structure achieves a large active area, while having a high responsivity and bandwidth.

**Figure 5:**
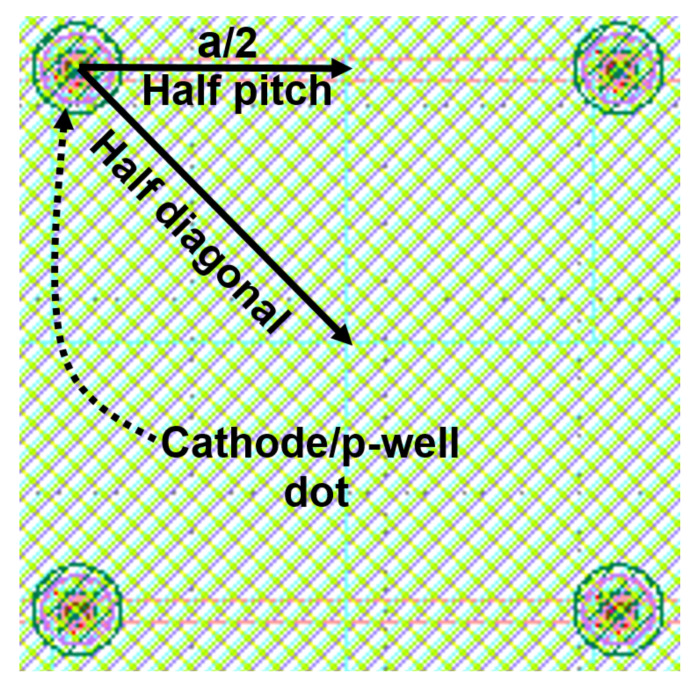
A zoomed top view of a cathode/p-well dot adjacent to other cathode/p-well dots in the array.

Based on this approach, the active area can be easily enlarged by expanding the cathode dot array. In fact, the active area can be enlarged by increasing the number of cathode dots arrays and also by increasing the distance between the cathode dots (i.e., the array’s pitch size). However, the pitch size of the array is an important parameter that affects the performance. Figure 5 is a zoomed top view of a cathode/p-well dot adjacent to other cathode/p-well dots in the array to visualize a half pitch and a half diagonal from the cathode center. The slowest response is expected if a photon is absorbed exactly in the center between four cathode/p-well dots, because this is where the longest drift distance along the silicon surface appears. This maximum lateral drift distance is $\frac{a}{\sqrt{2}}$, where *a* is the pitch of the n^+^/p-well dot array.

If the cathode dots are too far apart, there may not be a large enough electric field in the entire area between two cathodes to drive the photo-generated carriers to the cathode spot. Because, as discussed earlier, the electric field gradually drops by moving away from the center (multiplication region). Therefore, one cannot say that the entire area between two cathode/p-well dots is the detection zone, if the dots are too far apart. In addition, increasing the array’s pitch size results in a lower bandwidth. This is due to the fact that the frequency response is determined by the transit time of photogenerated electrons to reach the cathode. The transit time depends on the carrier drift distance as well as the drift velocity, which is proportional to the local electric field strength. Accordingly, the transit time is expected to be higher in MD-APD with a larger array pitch size because the carrier drift distance increases and, additionally, the intensity of the electric field decreases over the larger distance from the cathode dots.

On the other side, in the case where the distance between two neighboring cathodes is small, more cathode dots are required to achieve the same active area compared to that of the MD-APD with larger pitch sizes. Increasing the number of cathode dots leads to an increase in the MD-APDs capacitance due to the fact that the MD-APDs capacitance is the sum of the capacitance of all single dots in the array plus parasitic capacitance (of metal tracks). In addition, in the MD-APDs with small pitch sizes, more metal lines are needed to connect the cathodes together, leading to a larger parasitic capacitance. Furthermore, a larger area of the detection zone is covered by metal, which is an opaque material. Therefore this area is practically no longer called an absorption region because the incident photons are reflected by the metal before reaching the silicon. Accordingly, the pitch size of the array need to be optimized based on the requirements of the intended application.

**Figure 6:**
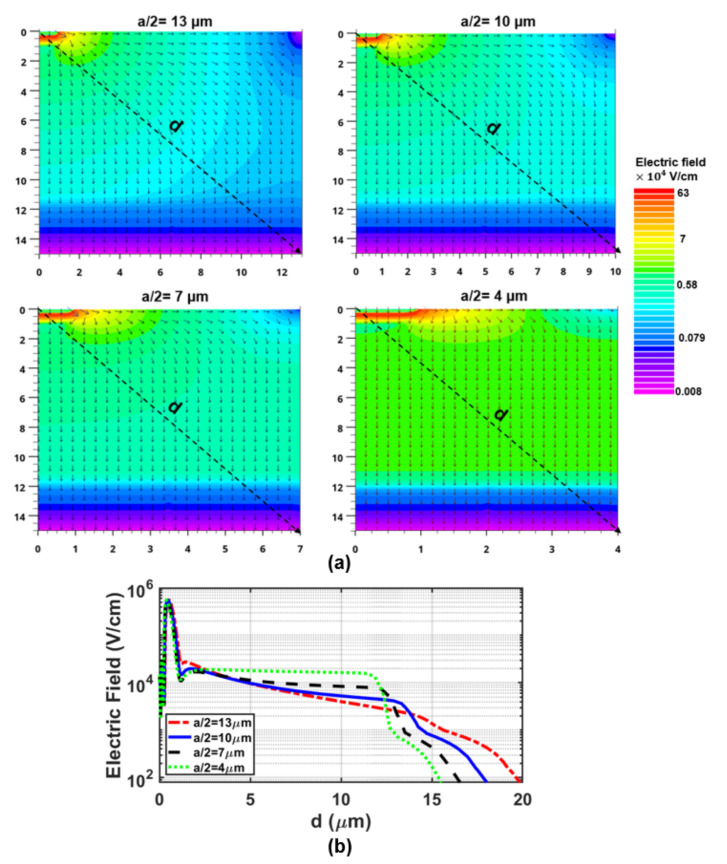
(**a**) 2d plot of the simulated electric field distribution in the 2d cross-section indicated in Figure 4b for different half pitch sizes *a*/2 of 13, 10, 7, and 4 μm at M = 40. (**b**) is the cross-section of the electric field along the dashed line (d) in sub-figure (**a**).

We used TCAD simulations with ATLAS to estimate an optimal array pitch size (a) of the MD-APD. It should be noted that we could not simulate the whole structure of the MD-APD because it requires a 3d simulation. Simulations with Cartesian coordinates are performed for a 2d cross-section indicated in Figure 4b, which contains a half-cathode dot at the corner of a half-pitch-wide region. Figure 6 illustrates the electric field distribution for different pitch sizes *a* of 13, 10, 7, and 4 μm. These chosen simulation regions are sufficient because of symmetry reasons and boundary conditions of the simulator.

**Figure 7:**
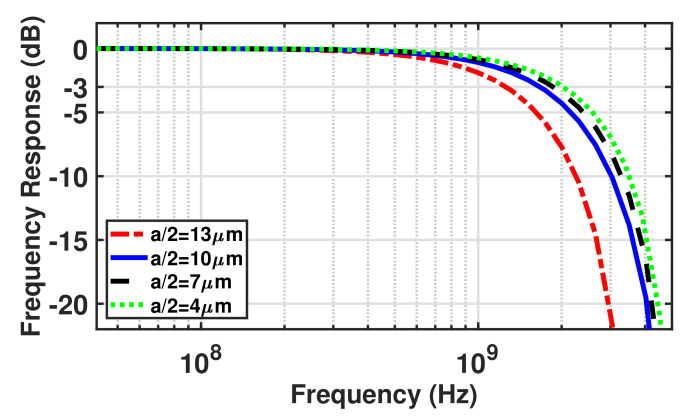
Normalized frequency responses at λ = 675 nm and M = 24 for different half pitch sizes of 13, 10, 7, and 4 μm, extracted from TCAD simulations.

According to the electric field vector arrows, the electric field extends along the surface and then down towards the substrate. It is visualized that the strength of the electric field distributed across the detection zone decreases when the pitch size increases. It can be seen in Figure 6b that the electric field strength at a “diagonal” distance *d* of 10 μm from the origin of coordinates in the structure with a half pitch size of 4, 7, 10, and 13 μm are 16.3 kV/cm, 8.4 kV/cm, 5.3 kV/cm, and 4 kV/cm, respectively. A higher electric field distributed across the detection region in smaller structures is expected to provide faster carrier drift transfer and thus lead to a higher bandwidth. Figure 7 shows the normalized frequency responses for the different half pitch sizes of 13, 10, 7, and 4 μm, at a wavelength of 675 nm and a gain of 40, obtained from TCAD simulations.

Figure 7 demonstrates that a higher bandwidth is achieved by shrinking the distance between cathode dots because of the electron drift time reduction due to the shortening of the radial carrier drift distance and an increase in the electric field strength distributed across the detection zone. It is shown that the structures with an *a*/2 of 13, 10, 7, and 4 μm achieve the bandwidth of 1.25 GHz, 1.7 GHz, 1.85 GHz, and 2 GHz, respectively. Taking a closer look at the dependence of the bandwidth on the cathode dots distance, it can be seen that reducing the *a*/2 from 13 μm to 10 μm increases the bandwidth from 1.25 GHz to 1.7 GHz, while further reducing the distance provides less bandwidth improvement. This is due to the fact that in smaller structures, the *a*/2 is smaller than the depth of the structure, and hence the vertical carriers’ transition from the deep depth (12 μm) limits the bandwidth, and therefore a further reduction of cathode dot distance has less influence on the bandwidth.

Now, the MD-APD can be designed according to TCAD simulation results and the above discussions. In the MD-APD design, a point that should be taken into account is that the bandwidth is limited by photo-generated carriers in the region between two adjacent cathode dots in the diagonal direction because it gives the longest drift distance (see Figure 5). The longest lateral drift distance along the silicon surface is $\frac{a}{2}\sqrt{2}=\frac{a}{\sqrt{2}}$. Here, the array pitch size of *a* = 14 μm is selected for the MD-APD structure in which the distance between two adjacent diagonal cathode dots is 20 μm. According to the simulated frequency response results for different distances of the cathode dots, it is expected that the MD-APD achieves a bandwidth between 1.7 GHz and 1.85 GHz, corresponding to *a*/2 = 7 μm and *a*/2 = 10 μm, respectively. Because carriers generated between two adjacent dots on the horizontal/vertical axis (*a*/2 = 7 μm) with a shorter drift distance and carriers generated in the area between two adjacent dots on the diagonal axis ($\frac{a}{\sqrt{2}}$ = 10 μm) with a longer drift distance contribute to the current flow (see Figure 5), so, the pitch *a* should be 14 μm. In the case where the pitch size of the array is *a* = 20 μm, the bandwidth reduction due to carrier transfer between diagonal cathodes is remarkable (see curve corresponds to *a*/2 = 13 μm from Figure 7) and thus the overall bandwidth significantly decreases. Therefore, an MD-APD shown in Figure 4 consisting of an array of 5 × 5 cathode dots with a pitch size of 14 μm can achieve a high bandwidth while providing an active area of 70 μm × 70 μm (exact active area: 5$a^{2}$−$a^{2}\left(1-\pi /4\right)\;=\;$4858 µm^2^). However, MD-APDs with a smaller pitch size can achieve a slightly higher bandwidth, but at the cost of reducing the active area and increasing the capacitance.

**Figure 8:**
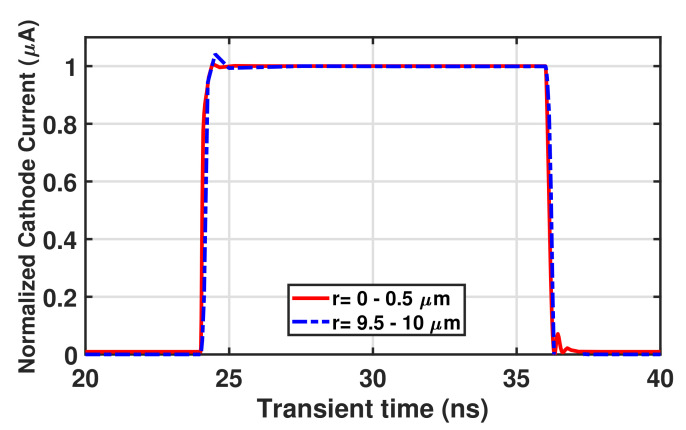
Transient response of MD-APD with a half diagonal 10 μm (pitch = 14 μm) at λ = 675 nm and M = 24, extracted from TCAD simulations.

Since there are different drift carrier path lengths in the MD structure, we should care about the timing jitter performance evaluation of the MD-APD. However, accurately calculating the jitter based on the transit time is challenging due to the variations in the electric field strength along the carrier’s drift path. Therefore, we have conducted transient simulations under different conditions to estimate the largest possible jitter in the MD-APD. In the first simulation case, we have limited the incident light to the center of the structure (r = 0 to r = 0.5 μm), and therefore only the photogenerated carriers in the center reach the cathode with the shortest drift length. As a result, the rise time of the transient response corresponds to the shortest carrier transit time. The transient response (Figure 8) for this condition shows a rise time of 160 ps. In the second simulation case, we have limited the light irradiation to the edge of the half diagonal of the MD diode (r = 9.5 μm to r = 10 μm; analogously to the frequency response simulations of the MD structure above), resulting only in the photogenerated carriers with the longest drift path reaching the cathode. Accordingly, the rise time of the transient response corresponds to the longest carrier transit time. A rise time of 240 ps is obtained from the transient response results in this condition (Figure 8). The difference between these two values provides an estimation of the maximum jitter in the MD-APD, which is approximately 80 ps.

## 4. Measurement Results

This section presents some key performance characteristics like capacitance, responsivity, and gain, as well as the bandwidth of the MD-APD, consisting of an array of 5 × 5 cathode dots with a pitch size of 14 μm fabricated in 0.35 μm CMOS technology. Measurements have been done on the wafer using a wafer probe station at 25 °C temperature regulated by a thermo chuck. A 675 nm laser source coupled with a multi-mode fiber with a core diameter of 62.5 μm feeds the light to the device under test. A Keysight B2987 electrometer was used to supply the voltage and measure the current.

### 4.1. Dark Reverse Characteristic and Capacitance

**Figure 9:**
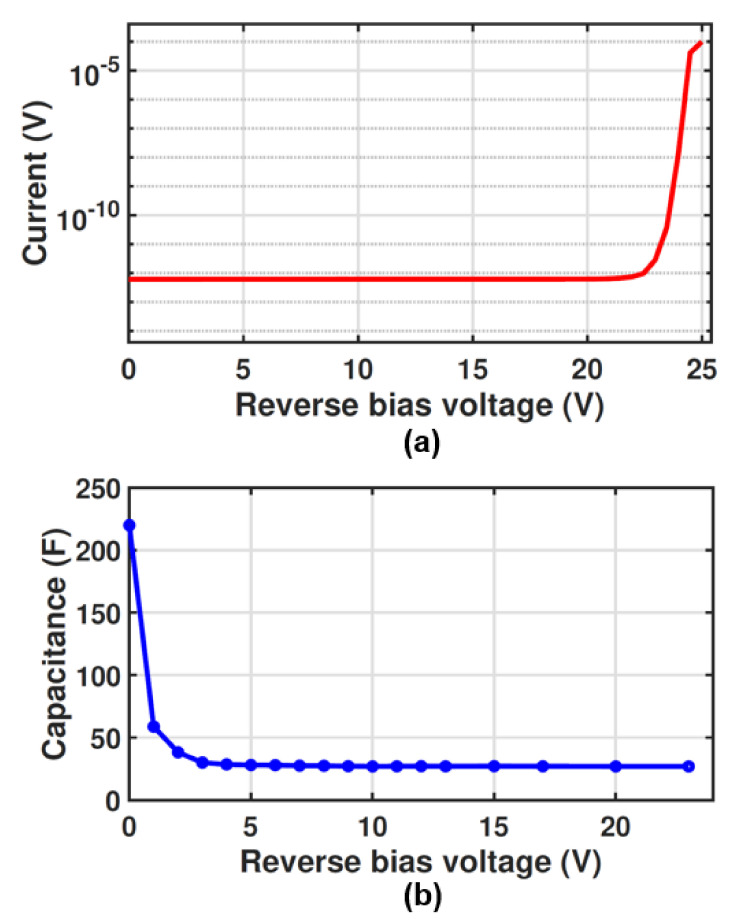
(**a**) Dark reverse characteristics and (**b**) capacitance of the MD-APD as a function of reverse bias voltage.

Figure 9a illustrates the dark reverse characteristics of the MD-APD as a function of the reverse bias voltage. The breakdown voltage of the MD-APD is 24.5 V (where the dark current reaches 1 μA) which is 0.7 V lower than that of the SD-APD. This is due to the fact that the electric field distribution in the multi-dot structure is different compared to that in the SD-APD.

Figure 9b illustrates the capacitance of the MD-APD, measured with an LCR meter Agilent 4284A at different reverse bias voltages with a frequency of 1 MHz and an ac amplitude of 100 mV. To subtract the effect of pad capacitance and measurement cables, the measurement was calibrated with an open structure (same pad size and metal track used for the MD-APD) on the same wafer.

The total capacitance of the MD-APD includes the capacitance of the p-n junctions of all single cathode dots and the parasitic capacitance of the metal tracks used to connect the cathode dots. The high capacitance of 220 fF at the zero voltage originates from the junction capacitance of the cathode dots which was estimated to be about 10 fF from the TCAD simulation result for SD-APD (see Figure 3b). As the reverse bias voltage increases, the capacitance decreases rapidly to 27 fF, of which 16.25 fF can be estimated as the junction capacitance of the cathode dots (25 × 0.65 fF), and the 10.75 fF left can be attributed to parasitic metal track capacitances. It is worth noting that the advantage of the low capacitance of this APD is significant for designing an optical receiver with a high data rate. Because in the design of the transimpedance amplifier (TIA), one critical factor limiting the bandwidth is the diode capacitance.

### 4.2. Responsivity and Gain

**Figure 10:**
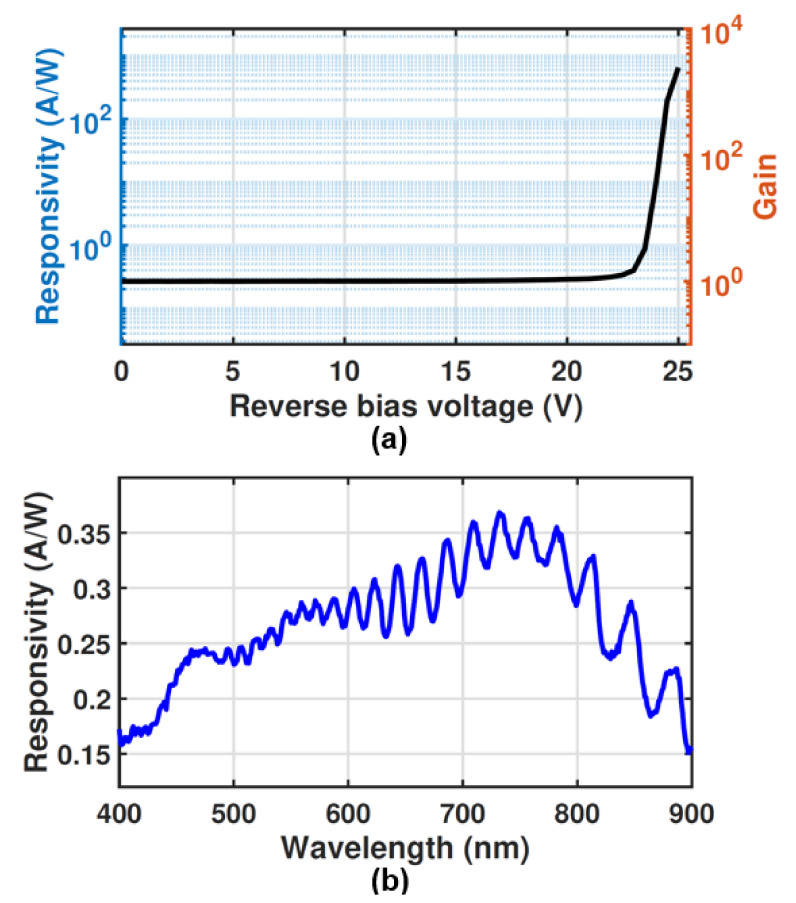
(**a**) Responsivity (left) and gain (right) of the MD-APD as a function of reverse bias voltage at λ = 675 nm and op = 200 nW. (**b**) Responsivity of MD-APD vs. wavelength (400 nm–900 nm) at M = 1 and op = 2 nW.

The photodetection characterization of the MD-APD has been done for λ = 675 nm. A fiber splitter is used to split up the light to feed it to the optical power meter from Thorlabs for power monitoring and to the fiber that feeds the light to the device under test. Based on the photocurrent characteristics, the gain and responsivity as a function of reverse bias voltage are obtained, where the dark current has been subtracted. Figure 10a shows the responsivity and gain as a function of the reverse bias voltage at an optical power (op) of 200 nW.

The APD shows a responsivity of 0.27 A/W at low bias voltages (Vop = 1 V) without gain (M = 1), which gives a quantum efficiency of about 50%. It is observed that the gain and responsivity are constant in a wide voltage range and then increase with a reverse bias voltage larger than about 20 V. At the operating voltage of 24 V, the responsivity and gain are 9.7 A/W and 36, respectively. However, the maximum achievable responsivity and gain depend on the optical power and decrease for higher optical power because of the saturation effect of the multiplication process at high optical powers. It should be mentioned that the diode can be biased at lower operating voltages, to work in the unamplified mode in the application where the optical power is high enough to be detected without amplification.

The spectral distribution of the unamplified responsivity (M = 1) is shown in Figure 10b. A monochromator (Digikrom CM110) is used to sweep the wavelength from 400 nm to 900 nm by steps of 1 nm. The light source is calibrated to provide a constant optical power of 2 nW over the spectrum using an optical power meter and a coupled variable optical attenuator. It can be seen that at λ = 675 nm and op = 2 nW, the responsivity of 0.27 A/W is achieved, which is as expected since the unamplified responsivity is independent of the optical power [25].

The λ-dependent fluctuations in the spectral responsivity originate from the formation of standing waves in the isolation and passivation stack covering the active region. Additionally, these layers lead to reflections for certain wavelengths that reduce the responsivity because not each incident photon is transmitted to the silicon (detection region). Accordingly, higher responsivities can be achieved if the silicon surface is covered by an anti-reflection layer that transmits almost all incoming photons into the silicon. Nevertheless, the spectral responsivity shows an average unamplified responsivity of 0.33 A/W in the spectral range between 700 nm and 800 nm, which is due to the thick detection zone as expected from the TCAD simulation results. Furthermore, the maximum unamplified responsivity of 0.37 A/W is achieved at the wavelength of 732 nm.

### 4.3. Frequency Response

**Figure 11:**
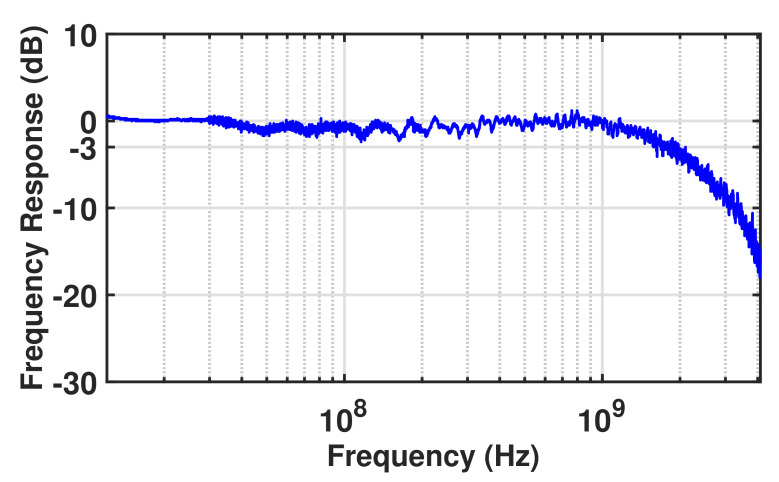
Normalized frequency responses at Vop = 24 V, op = 200 nW (λ = 675 nm).

Figure 11 illustrates the normalized frequency response of the MD-APD measured at the operating voltage of 24 V (R = 9.7 A/W and M = 36) and an optical power of 200 nW (λ = 675 nm) using a vector network analyzer (Rohde&Schwarz ZNB8).

The frequency response indicates a 3-dB bandwidth of 1.8 GHz, which is a promising value for reach-through-based APDs, because such structures show limited bandwidths due to a high carrier drift time in their thick absorption zone. Such a high bandwidth obtained for the MD-APD is because of the electron drift time reduction due to the large radius of the half-sphere high-field region throughout the structure. The measured 1.8 GHz bandwidth for the MD-APD with a pitch size of 14 μm demonstrates a good agreement between the simulation and the experimental results, as we expected a bandwidth between 1.7 GHz and 1.85 GHz from the simulation results.

## 5. Discussion

The research work presented in this study focuses on the development of a dot avalanche photodiode (APD) that overcomes the trade-off between the light-sensitive area and capacitance of planar APDs commonly used in optical communication systems. One of the main challenges with planar APDs is the need to decouple the light-sensitive area from the P/N-junction area to reduce the capacitance while maintaining the light-sensitive area. The proposed dot APD achieves this by using the lateral distribution of the electric field throughout the diode, enabling vertical and peripheral charge collection.

The innovative aspect of this work lies in enlarging the light-sensitive area by expanding the cathode dot array by offering low capacitance. The base for this is the very low capacitance of a single-dot APD at a large light-sensitive area. The performance of the multi-dot APD compared with various Si CMOS APDs shows a significant improvement in the responsivity-bandwidth product (R-BW product) of 17.46 $\frac{A}{W}\cdot$GHz, corresponding to the responsivity and bandwidth of R = 9.7 A/W and BW = 1.8 GHz, respectively. Additionally, the capacitance of the multi-dot APD is four times smaller than that of planar APDs, while maintaining a comparable active area. Furthermore, the multi-dot APD shows significant improvement in scalability and the ability to maintain its bandwidth and responsivity performance during up/down scaling.

It is worth emphasizing that the responsivity and frequency response of the MD-APD are independent of the array size but are affected by the array pitch size. This is due to the fact that the distribution of the electric field is independent of the number of cathodes in the array, but it changes with the change in the distance between the cathodes. However, the capacitance is proportional to the array size as the number of cathodes and metal connection length changes in different array sizes. In addition, as the drift path of carriers only depends on the array pitch size (i.e., shortest and longest paths) and is independent of the array size, the timing jitter is the same for different array sizes. Therefore, designing MD-APDs with different array sizes follows the same approach. This is one of the advantages of these structures, which makes it possible to easily expand the active area while maintaining the performance. These results demonstrate the superiority of the multi-dot APD over the state-of-the-art APDs and make it a promising candidate for a wide range of applications.

To better highlight, the contribution of this work over the state-of-the-art, the key performance parameters of the MD-APD are compared with various Si CMOS APDs and shown in Table 1.

## 6. Conclusions

The characterization of a CMOS-integrated dot avalanche photodiode is presented. It is shown that a hemispherically uniform high electric field is formed around the central cathode, which serves as the multiplication region, and a lower electric field penetrates radially throughout the diode to guide charge carriers from the entire diode volume towards the multiplication region. Accordingly, because of the high electric field distributed over the structure, the presented APD offers a large light-sensitive area while achieving high responsivity and bandwidth at long wavelengths due to its thick absorption zone and drift-based carrier transport. The active area of 70 μm × 70 μm is achieved with the MD-APD, consisting of an array of 5 × 5 cathode/p-well dots with a pitch size of 14 μm. However, the active area can easily be further enlarged by expanding the dot array (keeping the same pitch), without changing the bandwidth and the responsivity. A responsivity-bandwidth product (R-BW product) of 17.46 $\frac{A}{W}\cdot$GHz, corresponding to the responsivity and bandwidth of R = 9.7 A/W and BW = 1.8 GHz, respectively, is achieved for the MD-APD at λ = 675 nm and at an operating reverse voltage of 24 V. The 5 × 5 MD-APD offers the advantage of a low capacitance of 27 fF. 

## Figures and Tables

**Table 1:** Performance comparison of linear-mode APDs.

Parameters	Ref. [17]	Ref. [28]	Ref. [19]	Ref. [24]	Ref. [14]	Ref. [25]	This Work
Structure	n^+^/p-well RT-APD *	p^+^/n-well APD	n^+^/p-well RT-APD *	p^+^/n-well CA-APD ^⊕^	Double p-well/ n-well APD	n^+^/n-well EFLC-APD ^#^	n^+^/p-well MD-APD
Technology	0.35 μm	0.25 μm	0.35 μm	0.35 μm	45 nm	0.18 μm	0.35 μm
Active area	r = 43 μm	10 × 10 μm^2^	r = 30 μm	40 × 40 μm^2^	20 × 20 μm^2^	r = 19 μm	70 × 70 μm^2^
Capacitance	125 fF	-	≥100 fF	1 fF	-	≤1 fF	27 fF
Operating voltage	63 V	12.2 V	47 V	68 V	20.8 V	69 V	24 V
Optical power	500 nW	1 mW	1 μW	2 μW	100 μW	200 nW	200 nW
Wavelength	670 nm	850 nm	670 nm	830 nm	850 nm	675 nm	675 nm
Gain	50	16.7	20	43.9	23	80	36
Responsivity	20.5 A/W	0.2 A/W	7.4 A/W	13.17 A/W	0.56 A/W	32 A/W	9.7 A/W
Bandwidth	850 MHz	5.6 GHz	2.3 GHz	275 MHz	8.4 GHz	1.6 GHz	1.8 GHz
R-BW product	17.4 $\frac{A}{W}\cdot$GHz	1.1 $\frac{A}{W}\cdot$GHz	17.02 $\frac{A}{W}\cdot$GHz	3.6 $\frac{A}{W}\cdot$GHz	4.7 $\frac{A}{W}\cdot$GHz	51.2 $\frac{A}{W}\cdot$GHz	17.46 $\frac{A}{W}\cdot$GHz

* RT-APD: reach-through APD; ⊕ CA-APD: current assisted APD; # EFLC-APD: electric field line crowding APD.

## Data Availability

The data presented in this study are available on request from the corresponding author.
